# Software to facilitate and streamline camera trap data management: A review

**DOI:** 10.1002/ece3.4464

**Published:** 2018-09-06

**Authors:** Stuart Young, Johanna Rode‐Margono, Rajan Amin

**Affiliations:** ^1^ School of Life Sciences University of Nottingham Nottingham UK; ^2^ IUCN/SSC Asian Wild Cattle Specialist Group, c/o Chester Zoo Chester UK; ^3^ The North of England Zoological Society/Chester Zoo Chester UK; ^4^ Zoological Society of London London UK

**Keywords:** big data, camera trap data management, camera trapping, data annotation, data storage, ecological monitoring, metadata, standardization

## Abstract

Improving technology and increasing affordability mean that camera trapping—the use of remotely triggered cameras to photograph wildlife—is becoming an increasingly common tool in the monitoring and conservation of wild populations. Each camera trap study generates a vast amount of data, which need to be processed and labeled before analysis. Traditionally, processing camera trap data has been performed manually by entering data into a spreadsheet. This is time‐consuming, prone to human error, and data management may be inconsistent between projects, hindering collaboration. Recently, several programs have become available to facilitate and quicken data processing. Here, we review available software and assess their ability to better standardize camera trap data management and facilitate data sharing and collaboration. To identify available software for camera trap data management, we used internet searches and contacted researchers and practitioners working on large camera trap projects, as well as software developers. We tested all available programs against a range of software characteristics in addition to their ability to record a suite of important data variables extracted from images. We identified and reviewed 12 available programs for the management of camera trap data. These ranged from simple software assisting with the extraction of metadata from an image, through to comprehensive programs that facilitate data entry and analysis. Many of the programs tested were developed for use on specific studies and so do not cover all possible software or data collection requirements that different projects may have. We highlight the importance of a standardized software solution for camera trap data management. This approach would allow all possible data to be collected, enabling researchers to share data and contribute to other studies, as well as facilitating multi‐project comparisons. By standardizing camera trap data collection and management in this way, future studies would be better placed to guide conservation policy on a global level.

## 1 CAMERA TRAP STUDIES AS GENERATORS OF BIG DATA: THE NEED FOR EFFICIENT AND STANDARDIZED DATA MANAGEMENT

Camera traps—remotely triggered cameras to photograph wildlife—were not widely used to monitor or detect wildlife until toward the end of the 20th century when the technology was adopted by hunters to assist in tracking game. This new interest created a growing market for camera trap technologies, bringing costs down and increasing the variety of available equipment (Sanderson & Trolle, 2005). In addition to this, the move from film to digital cameras and advances in infrared sensors meant that ecological monitoring using camera traps became more feasible, accessible, and affordable (Burton et al., 2015; Kucera & Barrett, 2011). Now camera traps are used for a wide range of activities with different purposes such as biodiversity inventories, biodiversity and population monitoring, ecological and behavioral research, monitoring of human impact on ecosystems, and effectiveness of conservation interventions (Burton et al., 2015; O'Connell, 2015; Rovero, Tobler, & Sanderson, 2010; Rovero, Zimmermann, Berzi, & Meek, 2013; Rowcliffe & Carbone, 2008; Steenweg et al., 2016; Swann & Perkins, 2014). The large amount of information on camera trap technology, survey design, and statistical analysis shared in publications and conferences mirrors this interest (O'Connell, 2015). Numbers of published studies using camera traps have increased rapidly (Burton et al., 2015; Rowcliffe & Carbone, 2008), doubling every 2.9 years (Steenweg et al., 2016). Steenweg et al. (2016) estimated that camera traps have been employed at a minimum of 160,000 individual sites globally, with an average of 78 cameras used per study. Camera traps nowadays generate a vast amount of data (Rovero et al., 2010; Sanderson & Harris, 2013), with some studies recording up to 2.6 million images (McShea, Forrester, Costello, He, & Kays, 2016).

Once camera trap images have accumulated, a process starts that ultimately leads to data ready for analysis (e.g., see Harris, Thompson, Childs, & Sanderson, 2010; Krishnappa & Turner, 2014; Niedballa, Sollmann, Courtiol, & Wilting, 2016). First, images have to be retrieved from camera traps and stored securely. Secondly, files may need to be organized and labeled. Third—and often most time‐consuming—is image content identification and information management, which is also called image interpretation or annotation. Image annotation can be performed completely manually, but is becoming increasingly facilitated by technology, such as extraction of metadata from images for certain data (e.g., date and time). This whole process will be called “data management” hereafter. The final step of data analysis is covered elsewhere (e.g., O'Connell, Nichols, & Karanth, 2011; Rovero et al., 2013).

Several problems arise during camera trap data management. First, processing the massive amounts of data accumulated in camera trap studies requires a high amount of resources in terms of time and man power. This leads to a situation where data management rather than data collection is the limiting factor in the completion of studies (Barrueto, Clevenger, Dorsey, & Ford, 2013; Bubnicki, Churski, & Kuijper, 2016). Cataloging and classification of data often lag behind data acquisition, and sometimes a large amount of data remains unused and ultimately lost for science and conservation management (Harris et al., 2010). Further, such conservation and research projects are often tax‐payer funded through sources such as governmental grants, and uncataloged and analyzed data lead to a loss of public funds. In addition to this, such funds are often tied to a specific topic or research question, and so the cataloging and analyzing of images focus solely on the target species, for example. If all data were cataloged, however, then more conservation‐relevant outcomes in relation to funds could be generated, representing better “value for money”.

Secondly, as retrieval, storage and extraction of data from images is still mostly performed manually (except for standardized image metadata tags such as date, time, and label), human errors are introduced into data management and can lead to unintended data loss and incorrect data extraction from images (Krishnappa & Turner, 2014; Maydanchik, 2007; Sanderson & Harris, 2013).

Secondly, as retrieval, storage and extraction of data from images is still mostly performed manually (except for standardized image metadata tags such as date, time, and label), human errors are introduced into data management and can lead to unintended data loss and incorrect data extraction from images (Krishnappa & Turner, 2014; Maydanchik, 2007; Sanderson & Harris, 2013).

Finally, different choices in the process of data management, such as data coding, labeling of images, or data storage, may lead to a lack of accessibility, transparency, and inconsistency between projects. This prevents between‐project cooperation or effective data sharing (Harris et al., 2010; Meek et al., 2014; Rowcliffe & Carbone, 2008). Indeed, Chaudhary, Walters, Bever, Hoeksema, and Wilson (2010) found that across‐site comparisons and meta‐analyses are almost absent from the literature. Although two‐thirds of camera trap studies assessed by Burton et al. (2015) focus on multiple species as opposed to single species, often only a small proportion of these images contain the information required for a project, and a project may only annotate in respect to a limited amount of certain data variables, but not in others (Bubnicki et al., 2016; Wong & Kachel, 2016). However, in order to easily reuse the same dataset for other research areas (e.g., examining interspecific interactions and human‐wildlife conflict) or conservation management, all original images should be cataloged immediately, and in a transparent and consistent way (Burton et al., 2015; Chaudhary et al., 2010; Forrester et al., 2016). Having data accessible and in a standardized format makes it easier to share data and contribute to other projects which may have different focal species, as well as being able to compare sites (Wong & Kachel, 2016). A lot of research is conducted as short‐term projects or by individual researcher, so after their completion potentially important data may be lost for further future analysis (Hampton et al., 2013). The importance of sharing data in a consistent way does not only apply to the effective cooperation between research projects, but also within projects that involve large teams, often in different locations of the world, or projects relying on citizen science. In summary, standardized and transparent data management is essential to drive science and conservation management forward. This will enable researchers and practitioners to be successful in answering pressing ecological questions and guide conservation policy on a meaningful, possibly global, level (Meek et al., 2014; Wildlife Insights, 2017).

With this background situation, it is widely acknowledged that there is a requirement for a universal, user‐friendly, and standardized way to manage, store, classify, and share camera trap data (Sanderson & Harris, 2013; Barrueto et al., 2013; Forrester et al., 2016). Data management and processing facilitated by technology would decrease required resources, significantly decrease the risk of human error, and make sharing of data between and within projects easy.

With this background situation, it is widely acknowledged that there is a requirement for a universal, user‐friendly, and standardized way to manage, store, classify, and share camera trap data (Sanderson & Harris, 2013; Barrueto et al., 2013; Forrester et al., 2016). Data management and processing facilitated by technology would decrease required resources, significantly decrease the risk of human error, and make sharing of data between and within projects easy.

The aim of this paper was to give an overview of available programs for camera trap studies that facilitate efficient and standardized data management. We assessed whether the available software is suitable for the various needs of different camera trap projects and identified gaps that should be addressed in further developments of comprehensive software that meet the most common and important needs.

The data collection for this review was conducted from January to April 2017. To identify relevant software, we initially used the search term “camera trap data management” on Google and Google Scholar. This approach identified most existent software, while Agouti was identified by utilizing our professional contacts.

We conducted informal surveys (Barrueto et al., 2013) among colleagues and other researchers, enabling us to determine features and specifications that are perceived to be important to camera trap data management, as well as identify important data variables recorded during camera trap studies. Colleagues and researchers were identified using a snowball method, for example, each contact person was asked to suggest other experts in the field. In addition to experts’ advice, we included software characteristics identified by Ivan and Newkirk (2016), Niedballa et al. (2016) and Scotson et al. (2017). Using an existing set of camera trap images, we tested the available software, recorded each software's features and characteristics (Ivan & Newkirk, 2016; Niedballa et al., 2016; Scotson et al., 2017), and assessed its ability to record the data variables we had identified. If more features were found during this testing, we added them to the list and tested all software against them. Next, we compared all features between programs and to the potential needs of projects. Finally, we explored potentially useful features that have not yet been incorporated into available software, such as automatic subject recognition (He et al., 2016; McShea et al., 2016; Wang, 2014; Yu et al., 2013).

To simplify, we did not include applications that are used to primarily allow researchers to crowdsource image classification and annotation. These web applications allow citizen scientists to perform basic classification and annotation of images via a website (O'Connell, 2015), such as with Snapshot Serengeti (Hines, Swanson, Kosmala, & Lintott, 2015; Swanson et al., 2015) which uses the platform Zooniverse (Zooniverse 2017). Such applications are designed to be easy‐to‐use for nonexperts and thus do not usually include many of the features that may be necessary for a comprehensive standardized software.

## 2 CURRENTLY AVAILABLE CAMERA TRAP DATA MANAGEMENT SOFTWARE AND THEIR FEATURES

The vast amount of data being generated by camera trap studies around the world has led to an increasing number of programs to manage and process the generated image data. The number of available software increased from four programs identified in 2013 (Barrueto et al., 2013), five and seven, respectively, in 2016 (Ivan & Newkirk, 2016; Niedballa et al., 2016) and eight in 2017 (Scotson et al., 2017) to twelve published or otherwise available programs identified by us (Table 1). As long as no standardized and widely available software is produced and offered, the number of different, specifically tailored pieces of software may increase with the number of projects generating data.

**Table 1:** Software characteristics and features of tested camera trap data management programs, as well as image variables that tested programs are able to record and process**.** —represents “no”, or the absence of a feature

	Renamer & CamTrap^a^	ViXeN^b^	Aardwolf^c^	Camelot^d^	Snoopy^e^	Wild.ID^f^	Camera Base^g^	CPW Photo Warehouse^h^	eMammal[Link]	camtrapR^j^	TRAPPER^k^	Agouti^l^
General features												
Operating system	Windows	Windows, MacOS	Windows, MacOS, Linux	Windows, Linux	Windows, MacOS	Windows	Windows	Windows	Windows, MacOS	Windows, MacOS, Linux	Windows, MacOS, Linux	Windows
Installation requirements	.exe	.exe	mySQL	mySQL	mySQL	Java	MS Access	MS Access	Internet access	R	Internet access, see website	Internet access
Requires coding skills	Yes	–	–	–	–	–	–	–	–	Yes (R)	–m	–
Open source	Yes	Yes	Yes	–	–	–	–	–	–	Yes	Yes	– (but open to partners)
Web‐based	–	–	–	–	–	–	–	–	Yes	–	Yes	Yes
Data storage	Local	Local	Local	Local	Local	Local	Local	Local	Cloud	Local	Server–based	Cloud
Image storage capacity	Unlimited	c. 1,000,000	Unlimited	Unlimited	Unlimited	Unlimited	c. 2,000,000	c. 2,000,000	Unlimited	Unlimited	Unlimited	Unlimited
Functionality												
Automatic metadata import	–	–	Yes	Yes	Yes	Yes	Yes	Yes	Yes	Yes	Yes	Yes
Still/moving images	Still	Still	Still	Both	Both	Still	Both	Still	Still	Still	Both	Still
In‐built media viewer	Yes	Yes	Yes	Yes	Yes	Yes	Yes	Yes	Yes	–	Yes	Yes
Batch ID	–	–	Yes	Yes	Yes	Yes	Yes	Yes	Yes	Yes	Yes	Yes
Capture intervals	–	–	Yes	Yes	Yes	Yes	Yes	Yes	Yes	Yes	Yes	Yes
Filter/query data	–	Yes	Yes	Yes	Yes	Yes	Yes	Yes	Yes	Yes	Yes	Yes
Record active days	Yes	–	–	Yes	–	Yes	Yes	Yes	–	Yes	Yes	Yes
Automatic subject detection	–	–	–	–	Planned	–	–	–	Yes	–	–	–
Automatic species recognition	–	–	–	–	Planned	–	–	–	Yes	–	–	–
In‐built mapping	–	–	–	–	–	–	Yes	–	–	Yes	Yes	Yes
In app analysis	–	–	–	–	–	–	–	–	–	Yes	–m	–
Generation of standard reports	Yes	–	Yes	Yes	–	–	Yes	Yes	Yes	–	–m	–
Generate input files	.csv^n^; PRESENCE	–	–	.csv^n^; PRESENCE; R	Yes	–	.csv^n^; Excel; MARK; CAPTURE; DENSITY; PRESENCE; EstimateS	.csv^n^; Excel; MARK; PRESENCE; DENSITY; R	.csv^n^; PRESENCE, R	.csv^n^	.csv^n^	.csv^n^
Recordable data												
Camera make/model	Yesp	Yesp	Yes^p^	–	Yes	Yes	Yes	–	–	Yes	Yeso	Yes
Drop down species list	–	–	Yes	Yes	Yes	Yes	Yes	Yes	Yes	–	Yeso	Yes
Multiple species	–	Yes	Yes	Yes	Yes	Yes	–	Yes	Yes	Yes	Yeso	Yes
ID individuals	–	–	Yes	–	Yes	Yes	Yes	Yes	Yes	Yes	Yeso	Yes
Group size	–	–	Yes	Yes	Yes	Yes	Yes	Yes	Yes	Yes	Yeso	Yes
Age/sex classes per ID	–	–	Yes	Yes	Yes	–	Sex only	Notes	Yes	Yes	Yeso	Yes
Behavior per ID	–	–	Yes	–	–	–	–	–	–	Yes	Yeso	Yes
Weather variables	–	–	Yes	–	Yes	–	–	–	–	Yes	Yeso	–
Moon phase	–	–	Yes	–	Yes	Yes	–	–	–	Yes	Yeso	–
Sunrise/sunset	–	–	Yes	–	Yes	–	Yes	–	–	Yes	Yeso	–
Location variables	–	–	Yes	Yes	Yes	–	–	–	–	Yes	Yeso	–
Latitude/longitude	–	–	Yes	Yes	Yes	Yes	Yes	Yes	Yes	Yes	Yeso	Yes
Altitude	–	–	Yes	–	Yes	–	–	–	–	Yes	Yeso	Yes
Spatial (habitat) characteristics	–	–	Yes	Yes	–	–	–	–	–	Yes	Yes^p^	–
Support												
Multiple/shareable users	–	–	–	Yes	–	–	–	Yes	Yes	Yes	Yes	Yes
Crowd source IDs	–	–	–	–	–	–	–	Yes	–	Yes	Yes	Yes
Help/support	User manual	User manual	–	User manual, forum	User manual	User manual	User manual	User manual	Forum	Forum	Forum	User manual
Subscription fee?	–	–	–	–	–	–	–	–	Yes	–	–	Yes (to be discussed)
Reference	Harris et al. (2010); Sanderson and Harris (2013)	Ramachandran and Devarajan (2017)	Krishnappa and Turner (2014)	Hendry and Mann (2018)	Smedley and Terdal (2014)	Fegraus et al. (2011)	Tobler (2015)	Ivan and Newkirk (2016)	Forrester et al. (2013)	Niedballa et al. (2016)	Bubnicki et al. (2016)	

^a^G. Harris & J. Sanderson; smallwildcats.com/camera‐trap‐instructions ^b^K. Devarajan & P. Ramachandran; github.com/vixen‐project/vixen ^c^University of Oslo; github.com/yathin/aardwolf2 ^d^Flora & Fauna International – Vietnam; gitlab.com/camelot‐project ^e^R. Smedley; tulsasoftdb.com/snoopy ^f^TEAM Network; wildid.teamnetwork.org ^g^San Diego Zoo Global Institute for Conservation Research; http://www.atrium-biodiversity.org/tools/camerabase ^h^Colorado Parks & Wildlife; cpw.state.co.us/learn/Pages/ResearchMammalsSoftware.aspx^i^Smithsonian Institution; emammal.si.edu/ ^j^Leibniz Institute for Zoo and Wildlife Research; cran.r‐project.org/web/packages/camtrapR ^k^Mammal Research Institute of the Polish Academy of Sciences; bitbucket.org/trapper‐project/trapper‐project ^l^Wageningen University; agouti.eu. ^m^Users can program via application programming interface (API). ^n^Comma separated values. ^o^TRAPPER enables user to define any variable via API. ^p^Camera trap make and model (and other information) can be added using custom metadata.

During the testing phase, we checked all available programs against software characteristics they had to offer and the image‐related data variables that programs were able to record (Table 1). These features are discussed below, structured according to steps being involved in data management (Ivan & Newkirk, 2016).

### 2.1 Metadata import

The very first step of downloading and storing data from camera traps still needs to be performed manually. This preprocess can include the organization of directories, for instance. Some programs require a specific folder path and folder names to be able to process the data further (e.g., CamTrap), while others only require a basic folder structure with locations and camera trap ID. Another step may be the conversion of files, for example videos into web‐friendly formats if web applications are used. Extraction of metadata, such as date and time of image, is a crucial step of data management as it significantly decreases data entry errors. Some software are restricted to facilitating image metadata extraction (e.g., for date, time), but have no other application (Table 1). Other available metadata depends on the camera trap model; some models are able to record temperature, location, moon phase etc. It is important to note that while image standards such as Exif standardize certain metadata tags (e.g., time, date, and camera settings), other camera trap specific tags are not standardized (e.g., temperature, location). Therefore, a standardization of metadata by camera trap manufacturers would be of advantage (Forrester et al., 2016).

### 2.2 Facilitation of classification and annotation of images

Most available programs were developed for a specific project (but see e.g., Agouti, eMammal, TRAPPER), and accordingly, image classification is tailored to the respective project focus. Although some software is openly available and has been used for other projects, the tailored range of features and variables compromises the use by a wider audience. This is mainly because of a fixed, predetermined range of survey, camera trap location, and image variables that can be entered (Table 1). A range of variables are listed in Table 1. For instance, Wild.ID is highly suited for use on general biodiversity surveys, as it allows tagging of species in images; however, it does not offer the flexibility to gather more in‐depth data for each image (e.g., behavior). Camera Base was originally developed for tiger surveys and facilitates the (manual) identification of individuals in images in order to perform capture–mark–recapture analysis for population estimates. However, if the same image dataset were to be used for another research focus, for example, a project focussed on behavior instead of population size surveys, annotation of images would be difficult. One exception is for instance TRAPPER, an open‐source program designed to address a larger range of species and topics, and with an associated forum where users can discuss the use and further development of the program. Open‐source programs (Aardwolf, camtrapR, TRAPPER, ViXeN) are relatively rare, but may offer the opportunity to tailor image classification, survey characteristics, and other functions to specific projects. A disadvantage may be that this individualization of program features can lead to new problems in standardizing data management. Some projects may need an even more flexible design; ex situ research projects involving camera trapping may seek to study enclosure use (e.g., frequency of using certain enclosure parts in relation to visitor numbers) or very specific behaviors (e.g., stereotyping or aggression), while projects focussing on the monitoring of illegal human activities, for instance, may need to record those activities in relation to wildlife populations (e.g., presence and activity of people in images). In conclusion, the facilitation of image classification and annotation is extremely important and widely recognized in most programs, but can still be restricted to the specific variables targeted in a given project. A start for standardizing data management could be made by agreeing on certain standards within distinct camera trap communities (e.g., taxonomic, geographical, or thematic focus), as an immediate standardization of all processes for all current camera trap programs may be impractical and impossible.

Almost all programs have an in‐built media viewer that makes it easy to go through images in order to classify them. Usually, the images that can be uploaded into these viewers are “still images”, but a few programs allow “moving images” (videos) as well. One problem with videos is that metadata are usually not stored on the file; for example, original date and time may get overwritten during image storing processes. Videos however may yield more information, for example in behavioral research (Kuijper, Bubnicki, Churski, Mols, & van Hooft, 2015; Swinnen, Reijniers, Breno, & Leirs, 2014) and particularly in marine studies (Bond et al., 2012; Ebner et al., 2014). As a result, some programs (e.g., eMammal) allow loading a series of photos taken from the same event and classify it as one data point, effectively creating a low frame rate video (McShea et al., 2016). Other programs (Camelot, CameraBase, Snoopy and TRAPPER) are able to deal with video file types (e.g., .avi) directly.

Camera Base, camtrapR, and TRAPPER feature various mapping capabilities. Camera Base and camtrapR can generate simple maps of camera locations and species records within the programs, while camtrapR can generate shapefiles to use with GIS software (Niedballa et al., 2016), and CameraBase can generate custom lists for use with GIS. TRAPPER features an interface for mapping within the program, while the provision of an Application Programming Interface (API) means that there is functionality with other open‐source software such as QGIS (Bubnicki et al., 2016).

Most of the tested programs offer the ability to sort and query data according to assigned tags. This function can be used to explore images for specific data occurrences (behaviors, species etc.) or to filter images for use in communications or outreach. Further, this can allow researchers to classify certain photos that may need to be reassessed—for example Wild.ID offers a “certainty” field, allowing users to tag and filter images according to the confidence of their assessment.

### 2.3 Generating export files and automatic analyses

All software tested offer the generation of a range of export files that can be used by a variety of analysis software, such as MARK (White & Burnham, 1999), PRESENCE (Hines, 2017), DENSITY (Efford, Dawson, & Robbins, 2004), and R packages (e.g., unmarked [Fiske & Chandler, 2011], secr [Efford, 2015] or RMark [Laake, 2014]). Usually, these are comma separated values (.csv) or text (.txt) files, but some software also allow more specialized input files—for example, the generation of shapefiles for GIS programs by camtrapR. In‐built analysis of data is offered by camtrapR; however, knowledge of the coding language R is necessary for analysis.

### 2.4 Other characteristics

#### 2.4.1 Ease of use

Some programs tested are based on MS Access (Camera Base and CPW Photo Warehouse), and their manipulation requires respective skills. While MS Access skills are still relatively common, more complicated IT or programming skills are less widespread. The software TRAPPER is comprehensive, but it has one major limitation, which is acknowledged by its developers; the installation and maintenance require good IT knowledge for initial server configuration and updating of the source code (Bubnicki et al., 2016). camtrapR is a further open‐source program; however, its use requires knowledge and understanding of the R programming language. For a program to be used by a wide range of projects, especially projects led by local practitioners with limited IT and programming skills, the installation and tailoring of certain features to specific needs (e.g., variables needed for data collection) should be as easy as possible for the project administrator. In this respect, the inclusion of project configuration wizards into the software would be of great advantage.

#### 2.4.2 Web‐based programs and multi‐user options

In large multi‐site projects, various people may work from different locations on the same data set. In order to make work‐flow more effective, web‐ or cloud‐based software as well as the login of multiple users should be possible. Agouti, eMammal, and TRAPPER are web‐based, while several other programs allow multiple users. However, few programs so far allow the definition of the role of users; Agouti has two user levels, Project Investigator and other workers, that allow different activities. Assigning user roles can be very useful, for example, the set‐up of the study and data variables to be collected can only be set‐up by an administrator, while short‐term staff may just be allowed to have access to a certain part of the data processing. Where programs allow multiple users, a useful feature would be the ability to track changes in data annotation and image processing.

#### 2.4.3 Costs and memberships

Of the programs tested, eMammal has subscription and ongoing costs, leveled according to the number of images that need to be processed and stored, while for Agouti costs can be discussed. Both are among the most advanced and comprehensive of the programs assessed here. Assuming that maintenance and service costs increase with wider use, more comprehensive and widely available programs may be costlier for users (unless alternative means of funding, maintenance, and/or user service are used). All other programs tested are freely available, either by publicly available download or request.

## 3 FUTURE FEATURES

While technology and informatics in general is an extremely fast developing area, technology used in camera trap data management is moving relatively slowly. Often relevant technology exists, but a lack of interdisciplinary approaches as well as openness of technology means its application in camera trap data management is limited.

A less common, but useful, feature is automatic subject or movement detection, which is used by eMammal (He et al., 2016) and MotionMerkat (Weinstein, 2015). Generally, pixel values of a frame are compared to the distribution of pixels in the previous frame, adjusted for a variance, and either classified as background if no change occurred and foreground if a change occurred. In eMammal, this foreground is then extracted and displayed, which helps to identify small or well camouflaged animals that triggered the camera trap but may be easily overlooked when classifying through images (He et al., 2016; McShea et al., 2016). Following the automatic detection of the subject, the species can be manually identified.

It is recognized that species identification requires comprehensive taxonomic knowledge and is one of the most time‐consuming areas of work for nonexperts such as citizen scientists (He et al., 2016). Thus, a potential future feature that could further enhance data analysis and management is automatic species recognition. Various approaches for similar classification programs exist for other purposes, such as facial recognition software, and it is surprising that image classification in camera trapping is still performed manually (Yu et al., 2013). A few authors have attempted to develop species recognition processes, with varying success (He et al., 2016; McShea et al., 2016; Norouzzadeh et al., 2018; Villa, Salazar, & Vargas, 2017; Wang, 2014; Yu et al., 2013). Yu et al. (2013), for instance, developed a mechanism that extracts the foreground (the animal) from the background, analyses the features of the object, and finally classifies the images by a linear support vector machine algorithm. Using 7,000 camera trap images of 18 species from two different field sites, the authors report a classification accuracy of 82%. Wang (2014) reaches similar accuracies ranging between 77% and 87%, depending on the exact classification method and dataset, while He et al. (2016) reports lower levels of accuracy (34% and 38%; depending on method used). Most recently, Villa et al. (2017) and Norouzzadeh et al. (2018) have reported up to 98.1% and 96.6% accuracy, respectively, however, this drops significantly when incorporating an unbalanced dataset including uncommon species. Indeed, a disadvantage is that for each species, a high number of “practice” images must be available for the algorithm to learn, and this may not be possible for rare species (Norouzzadeh et al., 2018). Thus, a further development of automatic species recognition software would be extremely useful, for instance combined with the option to allow human assessments for low accuracy annotations or rare species.

A similar direction, but more specific to a single method, is computer‐assisted data extraction for population size estimations. Some software are able to match the same individuals based on natural individual markers, such as fur patterns, and use capture–mark–recapture methods to estimate population sizes (reviewed in Bolger, Morrison, Vance, Lee, & Farid, 2012). This approach is being developed for use in Snoopy (Smedley & Terdal, 2014). Further, McShea et al. (2016) aim to develop algorithms for eMammal that will extract information for use in Random Encounter Modeling, such as rate of movement (Rowcliffe, Carbone, Jansen, Kays, & Kranstauber, 2011), as well as biometric and behavioral data such as body size or group size. The integration of such algorithms into comprehensive software offering a range of different methods used in ecology seems sensible.

The burgeoning field of citizen science is being slowly integrated into camera trap data management software. While some specific websites and underlying processes exist that allow for basic image classification (e.g., SnapshotSerengeti, using Zooniverse), CPW Photo Warehouse and camtrapR are the only comprehensive programs that have specific features that facilitate the setting up of user IDs for crowd sourcing image classification.

In the future, high quality sound recording should be integrated into a camera trapping approach and respective software options developed, such as automatic recognition of sound patterns (Zaragozí, Belda, Giménez, Navarro, & Bonet, 2015). Soundscape ecology is defined as the collection of biological, geophysical, and anthropogenic sounds that emanate from a landscape and which vary over space and time reflecting important ecosystem processes and human activities and can be used to answer a variety of research questions (Pijanowski et al., 2011). Sound collection is more dynamic and may cover a wider range around the recording device in comparison with more spatially restricted image collection. By adding high quality audio sensors, a wider range of species may be detected (e.g., arboreal species), and the value of effort spent on data collection is maximized.

As a result of a more widely used comprehensive data management software, standardized data can be collected and used to answer spatially and temporally broader questions (Steenweg et al., 2016). To be able to make these data accessible, the development of software must go hand‐in‐hand with a data archive. Such databanks already exist for ecological research. Examples include EURODEER, a spatial database that stores shared roe deer (*Capreolus capreolus*) movement data and enables researchers to collaborate and produce better science (EURODEER, 2017). MOVEBANK is not restricted to a taxonomic group and archives animal movement data (Wikelski & Kays, 2010). The federated Wildlife Insights project has the potential for a similar platform for camera trap data (Wildlife Insights, 2017); for instance, the TEAM network that developed Wild.ID is working with this platform (Steenweg et al., 2016). Further, the Data Observation Network for Earth (DataONE) federation (Allard 2012) and its associated “nodes”, for example the Dryad Digital Repository (White, Carrier, Thompson, Greenberg, & Scherle, 2008), offer long‐term storage for scientific data. Using these repositories, standardized ecological data and metadata can be readily stored and accessed (Reichman, Jones, & Schildhauer, 2011), making them a valuable resource in standardizing camera trap data management.

## 4 CONCLUSIONS

Considering the increase of camera trap studies in the last few decades and the amount of data generated, it is surprising that so few comprehensive camera trap data management programs have been developed. Instead project‐specific software solutions are still dominant in the camera trapping community (Bubnicki et al., 2016). As these programs are not widely used across the camera trap community yet, they do not require high resources necessary for maintenance and service.

If databases are to be widely used and have impact on regional and global decision‐making, data management software should be easy‐to‐use, accessible, and in an open‐source format (Steenweg et al., 2016). So far, we have not been able to identify a single piece of software that would cover all possible needs that a variety of projects may have. Although we recognize that it may be challenging to develop a “one size fits all” solution (Barrueto et al., 2013), a comprehensive, yet flexible, program would be very beneficial in order to decrease the amount of resources needed to manage and classify camera trap data, decrease the amount of human error, and increase transparency and repeatability of projects. In order to serve this purpose, the respective program would include many of the features identified in this review (Table 1). Being open source may allow tailoring of the available features to the projects’ needs easily, but should do so in a transparent way, so data can still be shared. An interdisciplinary approach would be needed to facilitate the development of such a program, as the input of ecologists and computer programmers is needed. Such a program would complement the recommendation made by Scotson et al. (2017) that researchers should “adopt a standardized, nonproprietary, and transferable data storage format to store all camera trap data”.

Extending from recommendations by other authors to standardize camera trap methods and study designs (e.g., Ahumada et al., 2011; Scotson et al., 2017), we encourage a higher transparency in camera trap data management, processing, and storage in order to make datasets easily available for other purposes. This can be performed by developing more comprehensive and user‐friendly software. In agreement with Nichols, Karanth, and O'Connell (2011), we stress that the generation of big data is not the end purpose, but the understanding of ecological systems (science) and the effort to conserve and improve these ecosystems by informing decision‐making (conservation, management) should be the end purpose of these studies (Rowcliffe & Carbone, 2008; Steenweg et al., 2016).

## AUTHORS’ CONTRIBUTIONS

JRM and SY developed the concept of the review and collected information; all authors analyzed the findings. JRM and SY led the writing of the manuscript. All authors contributed critically to the drafts and gave final approval for publication.

## DATA ACCESSIBILITY

No data were collected.
